# Use of intravenous N-acetylcysteine in acute severe hepatitis due to severe dengue infection: a case series

**DOI:** 10.1186/s12879-021-06681-9

**Published:** 2021-09-20

**Authors:** D. M. D. I. B. Dissanayake, W. M. S. N. Gunaratne, K. W. M. P. P. Kumarihamy, S. A. M. Kularatne, P. V. R. Kumarasiri

**Affiliations:** 1 Teaching Hospital Peradeniya, Peradeniya, Sri Lanka; 2 Department of Medicine, Faculty of Medicine, University of Peradeniya, Peradeniya, Sri Lanka; 3 Department of Community Medicine, Faculty of Medicine, University of Peradeniya, Peradeniya, Sri Lanka

**Keywords:** Dengue fever, Severe dengue fever, N-acetylcysteine (NAC), Alanine aminotransferase (ALT), Aspartate aminotransferase (AST), Sri Lanka

## Abstract

**Background:**

Dengue fever is a common mosquito borne viral infection. Severe dengue fever associated severe hepatitis carries high mortality. Based on the beneficial effect of N-acetylcysteine (NAC) in paracetamol poisoning and non-acetaminophen induced liver failure, it is used in dengue fever associated hepatitis in clinical practice. We aim to study the reversal of liver enzymes with NAC in the setting of severe hepatitis due to severe dengue infection.

**Methods:**

A retrospective analysis was conducted on hospitalized 30 adults with severe dengue fever with severe hepatitis. These 30 patients had aspartate transaminase (AST) and alanine transaminases (ALT) more than 500 U/L and/or PT INR (prothrombin time and international normalized ratio) more than 1.5. They were treated with NAC infusion of 100 mg/h for 3 to 5 days.

**Results:**

The mean age of the group was 49.9 ± 11.46 years and 18 (60%) patients were males. Nineteen patients (63%) developed dengue shock. Of them 12 patients (40%) developed hepatic encephalopathy. Median AST on the day of administration of NAC was 1125 U/L interquartile range (IQR) 1653.25 while median ALT was 752 (IQR 459.25). There was a statistically significant reduction of both ALT (p = 0.034) and AST (p = 0.049) from day 1 to 4 after NAC infusion. Rise of platelet count between day 1 and day 4 also showed statistically significant difference (p = 0.011) but the reduction of prothrombin time and international normalized ratio (PT/INR) from 1 to day 4 did not show statistical significance difference. Mean duration of treatment with NAC was 3.61 ± 0.75 days while mean length of hospital stay was 6.2 ± 1.27 days. Only one patient died (3.3%). None of the patients reported adverse drug reaction due to NAC.

**Conclusion:**

Majority of patients demonstrated marked clinical and biochemical improvements and they recovered fully. We observed faster and significant recovery of liver enzymes following administration of NAC. Based on the above findings, this study provides preliminary evidence for the beneficial effect of NAC in severe hepatitis in dengue infection with greater survival benefits.

## Background

Dengue is a common mosquito borne viral infections in Sri Lanka. According to world health organization (WHO) more than half of world’s population in 128 countries are at risk of dengue infection and annually 390 million people are getting infected with the dengue virus. Sri Lanka experienced worst ever dengue epidemic in year 2017 with incidence rate of 866 per 100,000 population and that was a threefold rise compared to the previous year [1]. Dengue infection has a range of clinical manifestations. According to the WHO classification in 2009, dengue is classified as dengue fever (with or without warning signs) and severe dengue fever which includes severe plasma lekage, severe haemorrhage and severe organ impairment. Liver involvement is a common manifestation of dengue fever. Elevation of liver enzymes, aspartate aminotransferase (AST) and alanine aminotransferase (ALT) occur in 88% and 69% of cases respectively [2]. In majority of patients liver enzyme elevation do not cause hepatic dysfunction, but 4–7% of patients develop significant acute hepatitis with tenfold or more rise of aminotransferase levels [2, 3]. It carries a mortality rate of 50% due to complications such as encephalopathy, severe bleeding, renal failure and metabolic acidosis [4]. There are several mechanisms of severe liver involvement in dengue infection. i.e. hypoxic injury, direct viral invasion, immune mediated injury and secondary bacterial sepsis [5–7]. Liver injury can develop in prolonged dengue shock with plasma leakage but it is known to occur in dengue patients without evidence of plasma leakage [8]. Acute liver failure warrants costly novel interventions like molecular adsorbent recirculating system (MARS), which is shown to improve transplant free survival, [9] or urgent liver transplantation. But none of these methods are readily available in resource poor countries.

N-acetylcysteine (NAC) is an antioxidant agent which is known to restore hepatic glutathione reserve [10]. It is effective in acetaminophen induced hepatic failure. NAC is also proven to be effective in non-acetaminophen induced hepatic failure [11]. In addition to the restoration hepatic glutathione reserve, it improves the tissue oxygen delivery. In a prospective trial from Singapore, Lee et al. showed the effectiveness of NAC in non-acetaminophen induced acute liver failure where NAC was shown to improve transplant free survival in non-advanced grades of hepatic coma [12]. Use of intravenous NAC has not been studied extensively in dengue patients with severe hepatitis. A few case reports [13–17] and small case series [18] from Sri Lanka and Malaysia [19] showed the beneficial effect of NAC in dengue associated acute severe hepatitis. Manoj et al. reported a good outcome in a patient with dengue fever associated fulminant hepatic failure with advanced grade of coma i.e. grade 3 hepatic encephalopathy after treatment with intravenous NAC [17]. The beneficial effect of NAC in paediatric patients with dengue associated severe hepatitis was shown in case series by Senanayake et al. [20] and a case report by Lim et al. [21]. A recent study done in Thailand using mice has shown that NAC reduces dengue viral replication and oxidative damage to hepatocytes [22]. That study further showed that NAC helps to maintain antioxidant enzymes and redox balance in liver. Postulated mechanism is the induction of antiviral response via interferon signals. This study also showed the potential role of NAC as an antiviral agent. NAC is relatively a safe drug and majority of adverse drug reactions (ADR) are minor i.e. cutaneous anaphylactoid reactions, mild gastrointestinal reactions, mild respiratory reactions, central nervous reactions and cardiovascular reactions [23, 24]. Therefore, NAC therapy appears to be a safe treatment option for dengue fever associated severe hepatitis.

In the global literature, there are neither large case series nor randomized controlled trials on use of NAC in dengue patients with acute severe hepatitis. In 2017 during the outbreak of dengue in Sri Lanka, more than 4000 dengue patients admitted to Taching Hospital Peradeniya (THP) in the hilly Central Province and 40 patients of them were treated with NAC for severe hepatitis based on the elevation of transaminase levels. In this case series, we intended to study the effect of NAC therapy in dengue associated acute severe hepatitis. The main aim of the this study is to assess the improvement of liver transaminases, other clinical and laboratory parameters with NAC therapy in this group of patients.We also aim to study the survival outcomes and adverse reactions of NAC.

## Methods

### Study population

In the Teaching Hospital Peradeniya (THP), it is customary to maintain legible hospital records including monitoring charts of all dengue patients and to keep them in repository upon discharge in the hospital record room. In this study, we searched hospital database and retrieved medical records/ bed head ticket (BHT) of dengue patients who were treated with NAC. These patients were treated in the adult general medical wards and intensive care unit (ICU) of the THP from 1 of January to 31 of December 2017. According to the inclusion criteria, patients with confirmed dengue infection by positive NS 1 antigen who had liver transaminases (AST and ALT) > 500 IU/L and / or PT INR > 1.5 were included.In addition, these patients needed to fulfill completed intravenous NAC infusion of 100 mg per hour (administered in 500 mL of normal saline over 24 h) for 3–5 days treatment course depending on the time taken for clinical and biochemical improvement. Patients with pre-existing chronic liver cell disease (CLCD) or hepato-biliary disease were excluded. Pre-existing liver diseases were identified by scrutinizing past medical records, clinical examinations and ultrasound evidence of CLCD. In addition, dengue patients with evidence of other causes of acute hepatitis i.e. co-infection with viral hepatitis were also excluded from this study.

### Data collection

Of the 40 patients who were treated with NAC during the above period of time, data was collected in a formatted data collection sheet. Data which includes patient demographic details, clinical features and laboratory parameters were recorded. Laboratory parameters of serial liver biochemistry i.e. PT/INR, aspartate aminotransferase, alanine aminotransferase and serum bilirubin values were included. Evidence of hepatic encephalopathy and coma grade (1–IV) were recorded according to the West Heven criteria [25]. Data collection included serial white cell counts, haemoglobin, platelet counts, serum creatinine and ultrasonic evidence of plasma leakage also.In addition, data on duration of hospital stay, length of NAC treatment and survival status of patients were recorded. Details regarding NAC therapy was recorded i.e. treatment commencment, dose, duration of NAC therapy and adverse reactions.

### Statistical analysis

Continuous variables were expressed as mean and standard deviation (SD) or median with interquartile range (IQL). Categorical variables were expressed as percentages and frequencies. Statistical analyses was carried out using Statistical Package for Social Sciences (SPSS) version 17. Mann–Whitney test was used to compare AST and ALT values between day 1(D1) and day 4 (D4) as distribution of data showed right skewedness. i.e. skewness for AST = 5.296 and ALT = 4.247. Wilcoxon rank sign test was used to compare the difference of platelet count and PT/INR between D1 and D4 as sample sizes were small, although both variables showed normal distributions. Probability less than 0.05 was used as the cut off value for significance.

### Ethical approval

Ethical clearance was obtained from the ethics review committee, Faculty of medicine, University of Peradeniya. Informed consent was obtained from all subjects and for subjects who are under 18, from a parent. All methods were carried out in accordance with relevant guidelines and regulations.

## Results

**Fig. 1:**
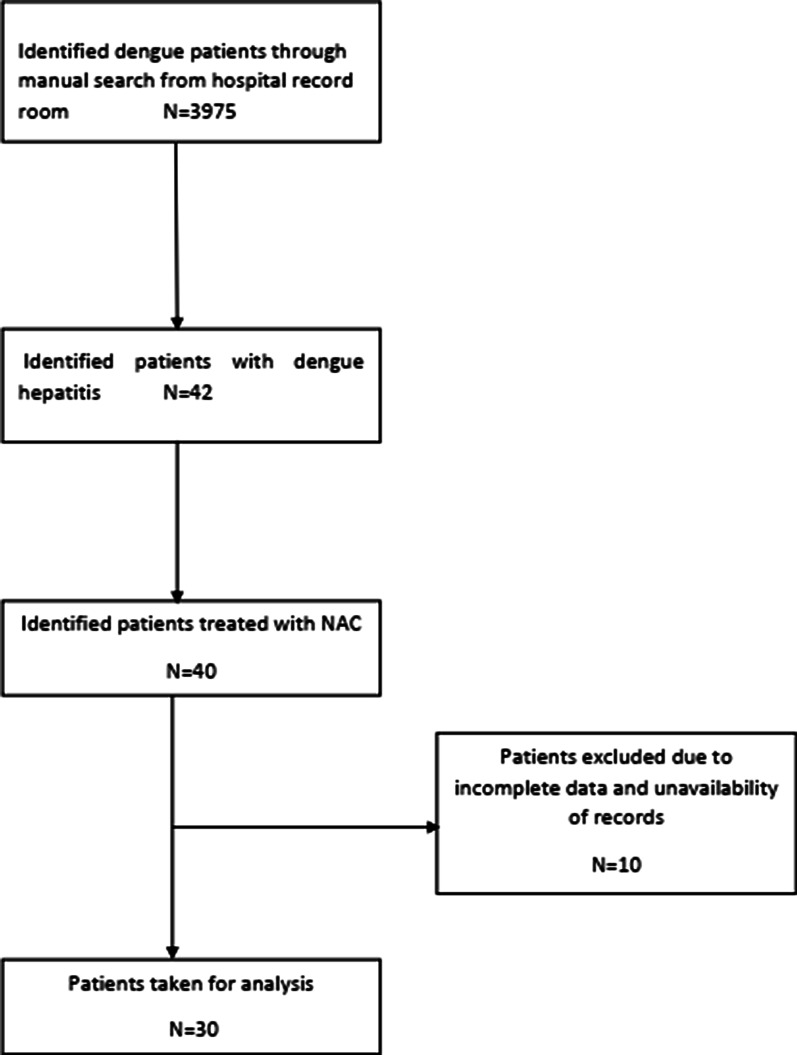
Data flow diagram

Total of 3975 dengue patient records were traced on dengue patients admitted to Teaching Hospital, Peradeniya between 1 of January and 31 of December 2017. Forty two patients fallen into the inclusion criteria were identified. Of 42 patients identified, 40 patients had NAC for acute severe hepatitis during their hospital admission. For the final analysis only 30 patients were included because some of the patient records were not available in the record room at the time of the study and some patient records did not carry all essential data for the analysis (Fig. 1).

Demographic characteristics and clinical characteristics are shown in Table 1. Of 30 patients, 18 (60%) were males. The age range was 16 to 65 years and mean age was 49.97 ± 11.46 years. On admission, the mean duration of fever was 6 ± 3.27 days. Nineteen patients (63%) developed manifestations of dengue shock during the hospital stay. Among those patients, 15 had compensated shock. Three patients experienced decompensated shock while profound shock was experienced by one patient. Hepatic encephalopathy was present in 12 patients (40%). Among 12 patients with hepatic encephalopathy, 10 patients developed grade 1 enephalopathy and one patient each developed grade 2 and 3 encephalopathy. Of 19 patients who had dengue shock, 12 patients experienced hepatic encephalopathy. Overt bleeding manifestations were seen in 4 (13%) patients in the form of menorrhagia or haematuria.

**Table 1:** Demographic details and clinical manifestations of patients

Characteristics	Number of cases (n = 30)
Sex	
Male	18 (60%)
Female	12 (40%)
Age (years)	
≤ 40	5 (16%)
41–50	7 (23%)
51–60	13 (43%)
61 ≤	5 (16%)
Clinical features	
Fever	30 (100%)
Back pain	30 (100%)
Headache	25 (85%)
Abdominal pain	27 (90%)
Reduced urine out put	5 (16%)
Bleeding	4 (13%)
Diarrhoea	2 (07%)
Skin flushing	30 (100%)
Conjunctival injection	4 (15%)
Icterus	11 (36%)
Right hypochondrial tenderness	30 (100%)

Investigations on the commencement of NAC treatment i.e. day one (D1) and day four (D4) are shown in Table 2. There was a statistically significant difference of ALT between D1 and D4 (p = 0.034). Difference between AST of D1 and D4 also showed a marginally statistically significant difference (p = 0.049).We analysed AST/ALT ratio D1 and D4 but it did not reach statistical significance (p = 0.711). Rise of platelet between D1 and D4 levels also showed statistically significant difference (p = 0.011). Reduction of PT INR between D1 and D4 did not show statistical significance (0.115).Serum bilirubin was not taken in to analysis because there was missing data in the medical records.

**Table 2:** Investigations on day 1 and day 4 following NAC treatment

	Day 1	Day 4	*p*
Hemoglobin g/L	125 ± 14.8	126 ± 14.8	
HCT %	40.07 ± 6.8	37.58 ± 2.57	
WBC × 10^9/^L	3.65 ± 1.26	6.31 ± 1.98	
Platelets × 10^9/^L	32.04 ± 28.09	57. 28 ± 54.32	0.011
INR	1.28 ± 0.17	1.14 ± 0.26	0.115
AST U/L	1125 (IQR 1653.25)	870 (IQR 1136.25)	0.049
ALT U/L	752 (IQR 459.25)	548 (IQR 762.00)	0.034
AST/ALT	1.859 (IQR 0.76)	2.042 (IQR 1.02)	0.711

Values for haemoglobin *WBC HCT* and *INR* are given as mean ± standard deviation. *AST ALT AST/ALT* are given as median with IQR indicated in brackets

Day 1 and Day 4 represents stating day and day 4 of NAC treatment respectively

p value indicates the difference of values between day 1 and day 4

*HCT* haematocrit; *WBC* white cell count; *ALT* alanine transaminase; *AST* aspartate transaminases; *PT INR* prothrombin time and international normalized ratio *IQR* interquartile range

**Fig. 2:**
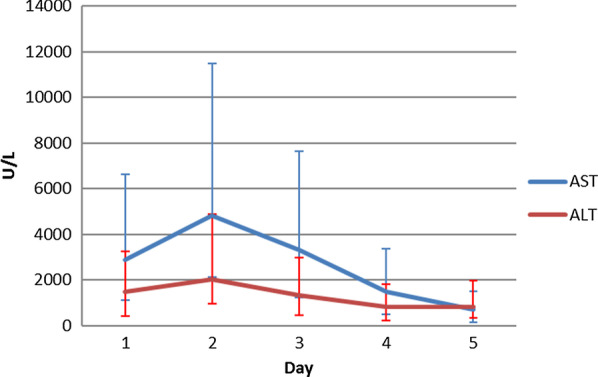
Trends of liver enzymes following treatment with NAC. Line diagram shows trends of AST and ALT plotted against days of treatment. AST is represented by blue line and ALT is represented by red lines. Both AST and ALT shows rise from day 1 to day 2. Both AST and ALT showed drop day 2. Reduction of AST and ALT was 47% and 62% between D1 and day D4 respectively. Difference between day 1 and day 4 were statistically significant i.e. p = 0.034 and p = 0.049 ALT and AST respectively. Error bars for AST and ALT are included in the graph. *ALT* alanine aminotranferase; *AST* aspartate aminotransferase; *NAC* N- acetylcysteine*; U/L* Units per Liter; *D1-* Day 1; *D4*- Day 4

Trends of AST and ALT are shown in Fig. 2.

None of the patients in this group reported any adverse drug reactions due to NAC. Mean duration of NAC treatment was 3.61 ± 0.75 days.The mean length of hospital stay was 6.2 ± 1.27 days. Of 30 patients only one (3.3%) died.

## Discussion

In this case series, we studied a series of 30 dengue patients with acute severe hepatitis who were treated with NAC. We found that NAC appears to be effective in reducing the liver transaminases particularly ALT in 4 days and it was a statistically significant reduction. In this series, 40% of patients had hepatic encephalopathy and they made recovery. The rise of platelet count was fast from day 1 to 4. Therefore, our study supports the NAC therapy in dengue infection complicated with acute severe hepatitis, but this finding needs to be further confirmed with powerful randomized controlled trials. However, NAC therapy has shown more survival benefit when compared to published outcomes, where dengue fever associated hepatic encephalopathy has 50% mortality rate [4]. When there is no facilities for emergency liver transplant and molecular adsorbent recirculating system (MARS) [26], the clinicians are more inclined to use NAC in dengue with acute sever hepatitis.

All 30 patients belonged to severe dengue fever category as they had plasma leaking and entered to critical phase of dengue. Among them, 19 patients (63.3%) went into develop shock state. Tweleve patients (40%) developed hepatic encephalopathy. These complications represent severe hepatic dysfunction and are near fatal. No doubt, all other supportive treatments would have helped to save lives of these patients. Dengue associated liver failure is known to occur in patients entering into dengue shock stage, but there are evidence that liver failure can occur even without the presence of dengue shock [4].The predominant dengue serotype during the outbreak in Sri Lanka in 2017 was DENV-2. This was a serotype change as DENV-1 was the predominant serotype in dengue epidemic reported from Sri Lanka in 2009 [16]. A study from Paraguay on patients aged less than 15 years found that frequency of liver involvement (measured in terms of increased transaminases) is similar among dengue serotypes 1,2,and 3[27].However, there are no data found on association between dengue serotype and dengue associated hepatitis in adult patients in the literature. In 2009, WHO introduced AST or ALT levels more than 1000 as a criterion for severe dengue, but study done by Lee LK et al. found that elevated level of aminotransferases may not discriminate between severe and nonsevere dengue fever [28].

AST/ALT ratio was studied in the setting of viral hepatitis associated acute liver failure and lower AST/ALT ratios are found to be associated with spontaneous survival [29]. In case of alcoholic hepatitis AST/ALT ratio is considered as marker of survival [30]. Further, in viral hepatitis B and C related cirrhosis, AST/ALT ratio is shown to have a prognostic capability and lower ratios are found to be related to the survival of patients [31].We evaluated the AST/ALT ratio in our patients with dengue associated acute severe hepatitis, but difference between day 1 and day 4 ratios did not reach a statistical significance. We observed a marked reduction of AST and ALT from D2 to D4 following administration of NAC. Habaragamuwa et al. [13] found that AST and ALT levels came down to 300 IU/L and 200 IU/L respectively on day 9 following treatment with NAC. Dalugama et al. [15, 16] also found that AST and ALT came down to 108 IU/L and 223 IU/L in 5 days with marked improvement in clinical and biochemical parameters. Some previous studies have shown that hepatic involvement in dengue fever takes 2 weeks to improve with reduction of AST and ALT to baseline with out any interventions [28]. In the retrospective analysis of Senanayake et al. in paediatric patients [17], both AST and ALT come down to 200 IU/L on day 8 following treatment with NAC. Faster recovery of AST and ALT following adminstration of NAC in these studies is similar to observations found in our case series. The rise of platelet from day D1 and D4 (p = 0.011) was also statistically significant. These observations may represent the action of NAC on recovery of dengue hepatitis. But further studies in the form of double blind randomized controlled trials are necessary to confirm the exact effect of NAC on recovery of liver functions, biochemistry and platelet count.

In this case series, 11 patients had early hepatic encephalopathy (grade 1 or 2) and one patient had advanced hepatic encephalopathy (grade 3). The case series published by Kumasena et al. [18] included 5 early stage patients (grade 1 and 2) and 3 late stage patients (grade 3 and 4). All 3 patients with advanced grade patients died while all 5 early stage patients survived. Habaragamuwa et al. [13] described a patient with early encephalopathy successfully treated with NAC. Dalugama et al. [15, 16] also reported two patients with dengue hepatitis in grade 1 hepatic encephalopathy. In contrast, Manoj et al. [17] reported a patient with dengue fever with massive haematemesis with stage 3 hepatic encephalopathy recovered completely following treatment with NAC and activated factor VII. This is one of the few patients with advanced hepatic encephalopathy recovered following treatment with NAC in the literature. In addition, recovery of a few patients with advanced encephalopathys was described in the case series by Tan et al. [19]. In a prospective double blind randomized controlled trial which was done on early non-acetaminophen induced liver failure, Lee et al. showed the efficacy of intravenous NAC in improving the transplant free survival. But it failed to show improvement in patients with advanced coma grades, which only benefited from emergency liver transplantation [10]. In the case series by Senanayake et al. [20] on paediatric patients, all 7 patients were in early hepatic encephalopathy and all survived following treatment with NAC. Lim et al. described [18], a 6-year-old child with dengue fever with advanced hepatic encephalopathy i.e. grade 3–4 fully recovered following treatment with NAC. Case reports by Manoj et al. [15] and Lim et al. [18] indicated a very important observation on the use of NAC in advanced hepatic encephalopathy. This would be a very important invention for countries like Sri Lanka if it is proven by powerful studies in the future.

In our case series, there was only one patient with advanced coma i.e. grade 3 and patient died despite treatment with NAC. But the patient presented 12 h after development of dengue shock and also had several comorbidities like diabetes mellitus, advanced diabetes nephropathy and advanced heart failure. He has previously undergone coronary artery bypass-grafting (CABG). He developed advanced coma with grade 3 encephalopathy with septicaemia and ultimately, died due to multi-organ failure following septic shock. Therefore, the death may not be directly related to dengue fever itself.

Regimens of intravenous NAC used differ among studies. In this case series the used regimen was infusion of 100 mg/h for 3–5 days. Dalugama et al. [15, 16] used the same regimen. Kumrasena et al. [16] and Abesekera et al. [12] used a bolus dose of NAC 150 mg/kg in 100 mL over 15 min followed by infusion for 3 days. In paediatric patients, Senanayake et al. [17] used 100 mg /kg for 24 h 1 to 3 doses and Lim et al. [21] also used the same regimen. In the case report of Habaragamuwa et al. [13] used NAC 100 mg/kg/day infusion for 5 days in an adult patient. Therefore, at present there is no consensus on treatment regimen of NAC for acute severe hepatitis in dengue patients. None of the patients in our case series report adverse drug reactions attributable to NAC. None of the patients above mentioned case reports or case series also developed ADR due to NAC. Hence, NAC appears as a safe treatment option to use in dengue fever associated severe hepatitis. Safety and efficacy of these treatment regimens needs to be established by powerful randomized controlled trials in the future.

Our study has some limitations. We were unable find age sex match controls in the hospital database. In addition, we were only able to collect a smaller sample for this case series as we had to exclude 10 patients. Some medical records/BHT misplaced in the hospital record room at the time of data collection and some patient records did not carry all essential data for analysis. As such, this case series gives descriptive analysis and lacks robust statics of comparison of groups.

## Conclusion

This case series provides preliminary evidence for the beneficial effect of NAC on recovery of liver enzymes with a greater survival benefits in patients with dengue fever associated acute severe hepatitis. In addition, it also provides some evidence for safety of NAC treatment in this setting.

## Data Availability

The datasets used and/or analysed during the current study are available from the corresponding author on reasonable request.

## Abbreviations

NAC: N-acetylcysteine
ALT: Alanine aminotransferase
AST: Aspartate aminotrasferase
IQR: Interquartile range
WHO: World Health Organization
PT INR: Prothrombin Time and International Normalized Ratio
DF: Dengue fever
MARS: Molecular adsorbent recirculating system
ADR: Adverse drug reactions
THP: Teaching Hospital Peradeniya
ICU: Intensive Care Unit
BHT: Bed head ticket
CLCD: Chronic liver cell disease
SPSS: Statistical Package for Social Sciences
SD: Standard deviation
HCT: Haematocrit
WBC: White cell count
DENV-1,2,3,4: Dengue virus types
